# Hydroclimate variability in the Caribbean during North Atlantic Heinrich cooling events (H8 and H9)

**DOI:** 10.1038/s41598-022-24610-x

**Published:** 2022-12-01

**Authors:** Yassine Ait Brahim, Matthew C. Peros, André E. Viau, Mercedes Liedtke, Jesús M. Pajón, Julio Valdes, Xianglei Li, R. Lawrence Edwards, Eduard G. Reinhardt, Frank Oliva

**Affiliations:** 1 International Water Research Institute, Mohammed VI Polytechnic University, Ben Guerir, Morocco; 2 Department of Environment and Geography, Bishop’s University, 2600 College Street, Sherbrooke, QC Canada; 3 Laboratory for Climate Change Research (LCC), Department of Geography, Environment and Geomatics, University of Ottawa, Ottawa, ON Canada; 4 Department of Paleogeography and Paleobiology, Museo Nacional de Historia Natural de Cuba, Obispo 61, Plaza de Armas, La Habana Vieja, CP: 10 100 Havana, Cuba; 5 Digital Technologies Research Centre, Data Science for Complex Systems Team M50, National Research Council Canada, 1200 Montreal Rd, Ottawa, ON K1A 0R6 Canada; 6 Institute of Earth Environment, Chinese Academy of Sciences, Xi’an 710061, China; 7 Department of Earth Sciences, University of Minnesota, Twin Cities, Minneapolis, MN USA; 8 School of Earth, Environment, and Society, McMaster University, 1280 Main Street West, Hamilton, ON L8S 4K1 Canada

**Keywords:** Geochemistry, Climate change, Palaeoclimate

## Abstract

We present a speleothem record from western Cuba, spanning the period 98.7–84.9 ka BP. Our record shows two distinctive periods of high δ^18^O corresponding to dry and/or cold periods during 85–87.6 and 90.2–93.1 ka BP, synchronous with Heinrich events 8 and 9 (H8 and H9). Hence, we provide the first proxy evidence of the local Caribbean climate response to H8 and H9. Interestingly, H8 is more pronounced compared to H9, which may be a local response to lower temperatures in the North Atlantic resulting in a weak AMOC and reduced deep water formation, therefore a stronger south shift of the ITCZ. Our data complement existing speleothem records from western Cuba which, collectively, provide a nearly continuous paleoclimate time-series spanning the last 100 ka BP, indicating a consistent response to millennial-scale events as dry and/or cooler conditions. The comparison with regional paleoclimate records reveals an anti-phased relationship with South America, caused by the southern movements of the ITCZ during millennial-scale events which lead to dry conditions in the Caribbean and a stronger South American Monsoon System.

## Introduction

The study of climate change from the Pleistocene to the present can contribute to a better understanding of the magnitude and rates of change of natural climate variability in the context of current global warming, especially in key study areas. In this context, climate and hydrological conditions in the tropical and subtropical Atlantic play a crucial role in the worldwide climate system, either as a response to changes in the Northern Hemisphere or as a driving mechanism for global climate conditions. The Caribbean is a highly interesting study region—located in the subtropics, influenced by climate processes of hemispheric interconnection including Pacific and Atlantic oceanic and atmospheric processes^1,2^. The competing influence of these modes of climate variability on tropical Atlantic climate is called the Tropical Atlantic Variability (TAV)^1,2^. The Caribbean region represents an important area to obtain information about past natural climate variability in the tropics and its relationship to climate forcing mechanisms. Climate change has also had a significant impact on the human history of the Caribbean region. For instance, it is believed that catastrophic droughts on the Yucatan peninsula contributed to the demise of the Maya civilisation^3–5^, and paleoclimatic research using speleothems has been important in helping to define the timing, spatial extent, and severity of the droughts that occurred at this time^6^. Therefore, it is essential to understand the factors influencing climatic variability in the Caribbean.

There are a small number of paleoclimate reconstructions for Cuba where most are limited to the Holocene or the Pleistocene/Holocene transition, using either sediment cores^7–10^ or speleothems^2,6,11^. Recently, Schielein et al.^12^ took fossil corals, from the terraces on the northern coast of Cuba, for dating using Electron Spin Resonance (ESR) and Uranium-series (U-Th) to determine the chronology of the Marine Isotope Stage (MIS) 5e coral reef formation. The age data shows one MIS 5e sea-level highstand around 126 ka. Furthermore, Warken et al.^13^ extended previous paleoclimate evidence for Cuba back to 96 ka using a speleothem record. This study combined stable isotopes of oxygen and carbon with trace element analysis. It was found that North Atlantic cold events were indeed recorded in the Caribbean, with abrupt cooling events (Henrich stadials), in particular, associated with relatively cold and/or dry conditions in Cuba^13^. This record showed that H1 was the driest and coolest interval of the whole record. During Greenland warm interstadials it was found that these produced relatively warm and wet conditions—especially during MIS 5. During the transition into the Holocene, this record shows a gradual change to generally warmer and wetter conditions. However, the speleothem record by Warken et al.^13^ is not continuous as this record shows a hiatus during the period ~ 93 to 81 ka. Thus, additional records are needed that cover longer time periods with high temporal resolution.

**Figure 1:**
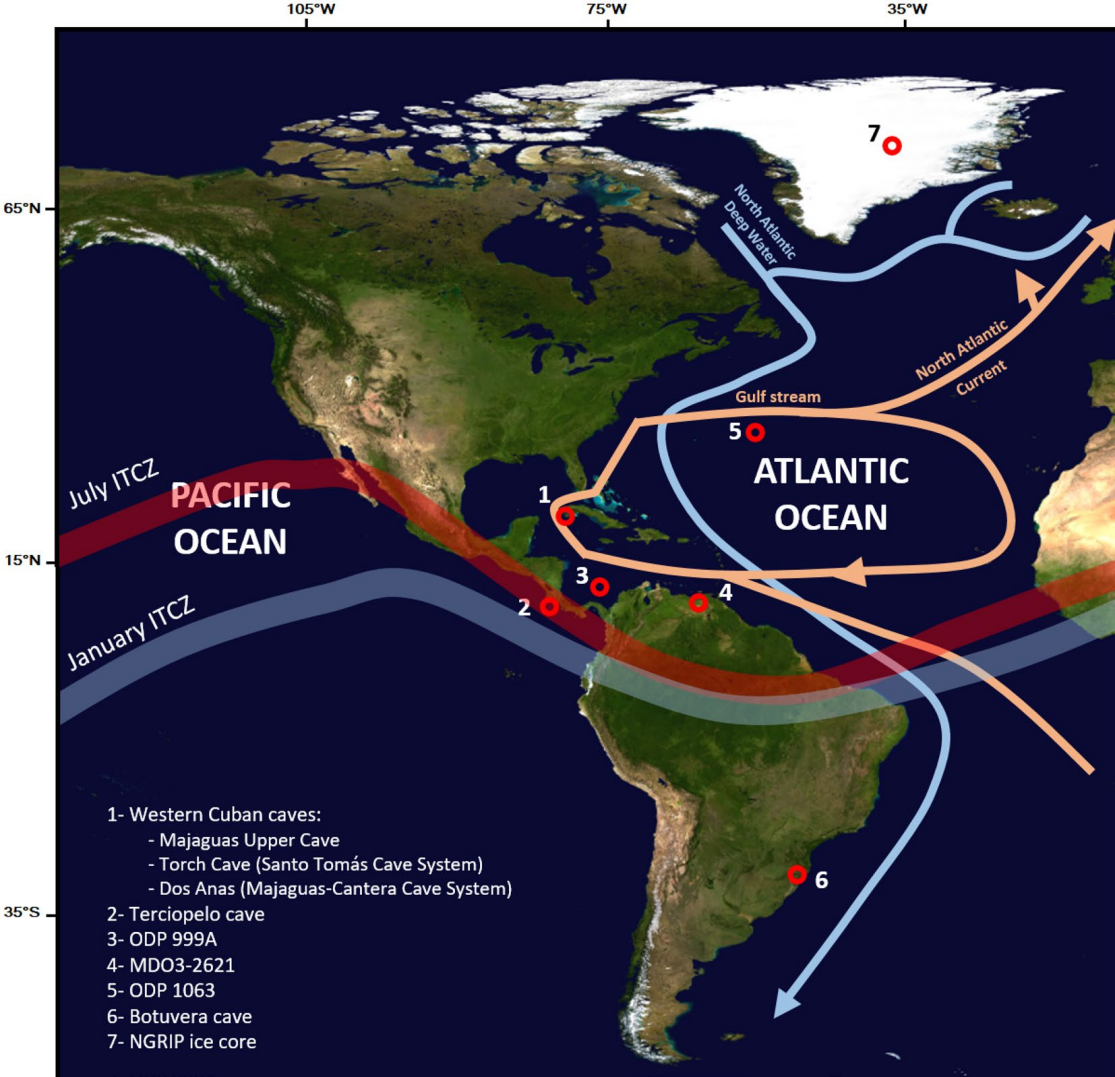
Map indicating the approximate locations of the most relevant sites discussed in this manuscript: (1) western Cuban caves, including the Majaguas Upper cave (this study), Torch Cave (Santo Tomás Cave System)^13^, and Dos Anas Cave (Majaguas-Cantera Cave System)^6^; (2) Terciopelo Cave, Costa Rica^15^; (3) Sediment core ODP 999A, Caribbean Sea^16^; (4) Sediment core MDO3-2621, Cariaco Basin^17^; (5) Sediment cores ODP 1063 and CDH19, Bermuda Rise^18–21^; (6) Botuvera cave, SE Brazil^22^; (7) NGRIP Ice core, Greenland^23–28^. The Atlantic Ocean circulation patterns and typical positions of winter and summer ITCZ are shown as well. The map was made using Adobe Illustrator.

In this study, we present a new high-resolution paleoclimate record from Cuba, in the Caribbean region, developed from a stalagmite (hereafter “MCS-01”), collected in the Salón de la Permanencia of the Majaguas Upper Cave that is a part of the Majaguas-Cantera Cave System in western Cuba (Fig. 1). The MCS-01 speleothem record covers the period 99 ± 3 to 84.9 ± 4.5 ka within MIS 5b, which has not been documented before in previous paleoclimate studies in the region. Analyzing the oxygen and carbon stable isotopes of MCS-01 will help fill these data gaps, and provide a more in-depth explanation for North Atlantic climatic events and their effects on sub-tropical regions. Due to the fact that speleothem archives are able to record continuous episodes of growth, their resolution can range on timescales from days to hundreds of thousands of years^14^. Speleothems can also provide excellent chronologies by virtue of their ability to be U-series dated^14^.

## Material and methods

### Stalagmite sample and regional setting

The stalagmite MCS-01 was collected in the “Salon de la Permanencia” (bedroom hall) of the Majaguas Upper Cave, which is part of the Majaguas-Cantera Cave System (22° 23′ N, 83° 58′ W) (Fig. 1). The Majaguas-Cantera Cave system is found in Pinar del Rio Province, which is located on the western side of the island. Pinar del Rio Province is characterized by one of Cuba’s main mountain ranges—the Cordillera de Guaniguanico. This mountain range is divided into the Eastern Sierra del Rosario and the Western Sierra de Los Órganos. The Pinar del Rio Province has a moderate subtropical climate with two seasons: the dry season from November to April and the humid season from May to October. Pinar del Rio is classified as “Am or tropical monsoon climate” according to the Koppen-Geiger climate classification^29^, with the average annual temperature being 24.9 degrees Celsius and the average rainfall accumulation is 1353 cm according to the ECMWF Data over the period 1991–2021.

The Majaguas-Cantera cave system is located in the Sierra de San Carlos karst area, which is part of the Sierra de los Órganos mountain range. The system has a total length of approximately 35 km with 10 cave levels of caverns ranging from 50 to 290 m elevation^30^. The ground surface over the cave is covered by thick vegetation and soil.

**Figure 2:**
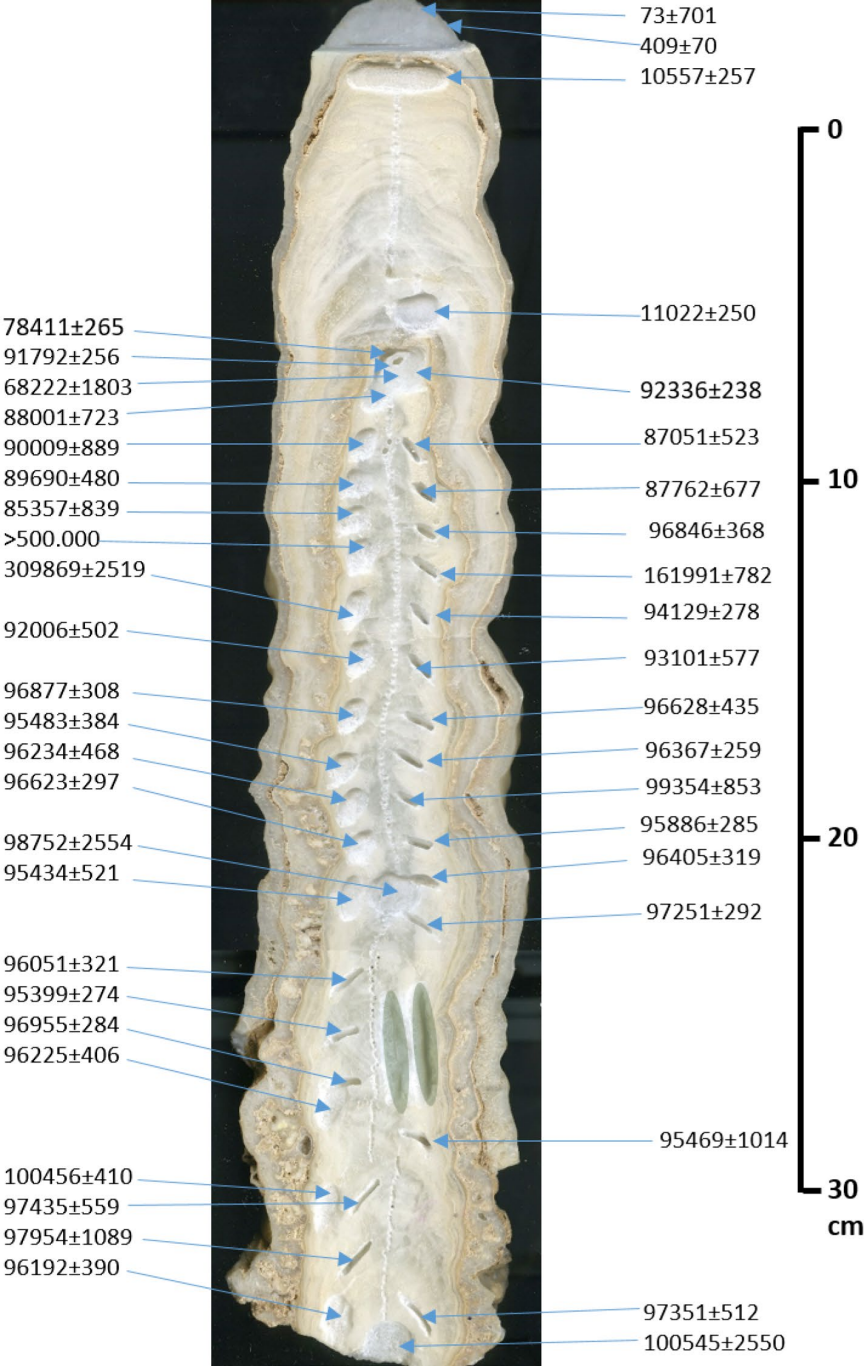
Stalagmite MCS-01 indicating the location of U–Th dating samples, with ages reported in B.P., and the approximate sample positions for the X-Ray Diffraction (green shading).

MCS-01 developed at 110 m above sea level, 200 m under the ground surface. The MCS-01 sample (Fig. 2) was cut down its central growth axis using a Target t*e*lematic 14″ diamond blade with a continuous rim and polished to show the internal structural details of the stalagmite. X-ray Diffraction (XRD) analyses were performed on two samples from MCS-01 using a Rigaku Ultima IV Diffractometer in the X-ray Core Facility at the University of Ottawa to confirm the nature of the carbonate material (i.e. calcite or aragonite).

### U-Th chronology

Calcite samples were taken from one-half of the speleothem along visible growth layers using a hand-held Dremel Rotary tool with a 0.3 mm diamond tip drill bit for U-Th radiometric dating. The positions of the preliminary samples were based on their proximity to potential hiatuses. The preliminary U-series measurements for age determination were performed at the Radiochronology Laboratory of the GEOTOP-UQAM-McGill Research Center, Quebec, Canada, whereas further U-dating was carried out at the Isotope Laboratory of the University of Minnesota, Minneapolis, MN, USA.

Regarding the analytical procedure followed at the Radiochronology laboratory of the GEOTOP-UQAM-McGill Research Center, 1 g of subsample powder was dissolved using nitric acid in a Teflon™ beaker into which a weighed amount of calibrated mixed spike ^233^U–^236^U^229^Th had been placed and evaporated slowly to dryness. After the dissolution, around 10 mg of the iron carrier was added and the solution was then left overnight for spike-sample equilibrium. U and Th were co-precipitated with Fe(OH)_3_ by adding ammonium hydroxide drop by drop until reaching pH 8–9. The precipitate was recovered by centrifugation and washed twice with deionized water, then dissolved in 6 N HCl. U–Th separation was performed on a 2 mL AG1X8 anionic resin volume. The thorium fraction was recovered through elution with 6 N HCl, and the U and Fe fraction, with water. The U fraction was purified on a 0.2 mL U-Teva™ (Elchrom industry™) resin volume. Fe was eluted with 3 N HNO_3_ and the U fraction with 0.02 N HNO_3_. After drying, thorium purification was carried out on a 2 mL AG1X8 resin in 7 N HNO_3_ and eluted with 6 N HCl. U–Th measurements were performed using a multi-collector inductively coupled plasma mass spectrometry Nu instrument™. ^236^U–^235^U–^234^U–^233^U and ^232^Th–^230^Th–^229^Th were measured on the ion counter (IC0) in peak switching mode for uranium and thorium isotopes, respectively. Knowing ^236^U/^233^U of the spike, mass bias corrections in the atomic mass unit (amu-1) were calculated and used to correct measured ratios between U isotopes and between Th isotopes^31^.

At the Isotope Laboratory of the University of Minnesota, uranium and thorium U/Th analyses were performed using a Neptune Multi‐Collector Inductively Coupled Plasma Mass Spectrometer (MC-ICPMS) separation procedure described by Edwards et al.^32^. For this, 0.2 g of carbonate subsamples were drilled. All errors are reported as two standard deviations (2σ). Standard chemistry procedures were used to separate U and Th. A triple-spike (^229^Th–^233^U–^236^U) isotope dilution method was employed to correct instrumental fractionation and determine U–Th isotopic ratios and concentrations. The instrumentation, standardization, and half-lives are reported in Cheng et al.^33,34^. All U–Th isotopes were measured in peak-jumping mode on a MasCom multiplier placed behind the retarding potential quadrupole. We followed procedures to characterize the multiplier similar to those described in Cheng et al.^33^. Uncertainties in U/Th isotopic data were calculated offline, including corrections for blanks, multiplier dark noise, abundance sensitivity, and spike composition. ^230^Th ages were corrected using an initial ^230^Th/^232^Th atomic ratio of 4.4 ± 2.2 × 10^−6^. The values are for material at secular equilibrium with respect to the bulk earth ^232^Th/^238^U value of 3.8. The U and Th decay constants are reported in Cheng et al.^34^.

### Stable isotope analyses

Samples for stable isotope analysis (oxygen and carbon) were taken using the hand-held Dremel Rotary tool fitted into a Dremel Rotary Tool Workstation Drill Press. The sample was placed on a velmex tree ring counter, in order to accurately sample the speleothem along the growth axis, where ~ 700 to 800 μg of the sample was collected. Stable isotope samples were processed at the University of Ottawa’s Ján Veizer Stable Isotope Laboratory.

Oxygen and carbon isotope values (ẟ^18^O and ẟ^13^C) were measured for 363 calcite samples drilled at discrete 1 mm intervals down the growth axis of the stalagmite. Approximately 500–700 μg of calcite was weighed for each sample and transferred to the exetainer for analysis. Stable isotopic analyses were conducted at the Ján Veizer Stable Isotope Laboratory, where 0.1 mL of degassed anhydrous phosphoric acid was added to the exetainer, while on its side. Once all vials had acid added, the exetainers were flushed and filled with UHP helium off-line for 4 min at a rate of 60–70 mL/min. Following this, the vials were then immediately placed upright and in the heating block of the GasBench, and left to react for 24 h. Regular analysis of the CO_2_ headspace was followed, using a Thermo Finnigan Delta Plus XP IRMS. The isotopic composition of the CO_2 (g)_ for each sample was measured relative to the international reference, the Vienna Pee Dee Belemnite (VPDB), using the conventional “δ-per mil” notation. The analytical precision of this instrument is + /− 0.15‰.

## Results and discussion

### Age model

A total of 44 U/Th dating analyses were performed on calcite samples from MCS-01, among which 5 preliminary dating analyses were carried out at the Radiochronology laboratory of the GEOTOP-UQAM-McGill Research Center (Table 1), and then 39 additional analyses were added in the Isotope Laboratory of the University of Minnesota (Table 2). Indeed, age reversals due to potential aragonite-calcite age discordance^35^ have already been shown in Western Cuba stalagmites^6^.The XRD results from the basal part of the stalagmite show that MCS-01 is composed of calcite. However, 11 major outliers were detected by the StalAge algorithm, and therefore excluded from the age model since their age and uncertainties are significantly far from the stratigraphic order (e.g. by several thousands of years). Another verification method that was adopted in the age dating of samples below and above each major outlier that could not be well dated to confirm them.

**Table 1:** U/Th dating results from the Radiochronology laboratory of the GEOTOP-UQAM-McGill Research Center.

Sample Number	Depth from Top (cm)	^238^U (ppb)	^232^Th (ppb)	^234^U/^238^U	(^230^Th/^234^U)	(^230^Th/^238^U)	(^234^U/^232^Th)	(^238^U/^232^Th)	(^230^Th/^232^Th)	230Th/U age ka (BP)	(^234^U/^238^U)
MCS-Top	1.6	128.60 ± 1.1	0.13 ± 0.0	1.32 ± 0.0	0.004 ± 0.0	0.005 ± 0.0	3881.1 ± 104	2940.7 ± 80.8	14.55 ± 2.5	0.41 ± 0.1	1.32 ± 0.0
MCS-4	2.6	123.95 ± 1.1	1.53 ± 0.0	1.33 ± 0.0	0.093 ± 0.0	0.123 ± 0.0	328.5 ± 2.6	247.3 ± 2.5	30.51 ± 0.6	10.56 ± 0.3	1.34 ± 0.0
MCS-3	9.3	125.82 ± 1.2	1.32 ± 0.0	1.33 ± 0.0	0.097 ± 0.0	0.129 ± 0.0	385.5 ± 3.1	290.3 ± 3.0	37.30 ± 0.7	11.02 ± 0.2	1.34 ± 0.0
MCS-2*	10.9	192.97 ± 1.8	8.64 ± 0.2	1.46 ± 0.0	0.479 ± 0.0	0.699 ± 0.0	99.5 ± 2.1	68.2 ± 1.5	47.70 ± 1.2	68.22 ± 1.8	1.56 ± 0.0
MCS-7	25.7	115.60 ± 0.6	1.05 ± 0.0	1.47 ± 0.0	0.621 ± 0.0	0.912 ± 0.0	494.2 ± 7.6	336.8 ± 4.2	307.11 ± 5.2	98.75 ± 2.6	1.62 ± 0.0
MCS-1	38.35	115.07 ± 0.6	0.87 ± 0.0	1.45 ± 0.0	0.628 ± 0.0	0.912 ± 0.0	585.0 ± 3.9	403.1 ± 3.0	367.39 ± 5.5	100.55 ± 2.5	1.60 ± 0.0

*Refers to dating results that were not used in the age model. B.P. stands for “Before Present” where the “Present” is defined as the year 1950 A.D.

**Table 2:** U/Th dating results from the Isotope Laboratory of the University of Minnesota.

Sample Number	Depth from top (cm)	^238^U	^232^Th	^230^Th /^232^Th	δ^234^U*	^230^Th/^238^U	^230^Th Age (year)	^230^Th Age (year)	δ^234^U_Initial_	^230^Th Age (year BP)
		(ppb)	(ppt)	(Atomic × 10^–6^)	(Measured)	(Activity)	(Uncorrected)	(Corrected)	(Corrected)	(Corrected )
MCS-6	0.55	128.4 ± 0.2	5654 ± 114	5 ± 0	300.0 ± 2.7	0.0134 ± 0.0003	1132 ± 23	143 ± 701	300 ± 3	73 ± 701
M1	10.2	317.2 ± 0.4	2429 ± 49	1646 ± 33	441.7 ± 1.8	0.7645 ± 0.0014	78,626 ± 245	78,482 ± 265	551 ± 2	78,411 ± 265
M43*	10.4	129.4 ± 0.1	909 ± 18	2011 ± 40	449.0 ± 1.4	0.8567 ± 0.0012	91,992 ± 239	91,863 ± 256	582 ± 2	91,792 ± 256
M44*	10.7	145.8 ± 0.1	566 ± 11	3644 ± 73	445.7 ± 1.4	0.8576 ± 0.0011	92,478 ± 233	92,407 ± 238	578 ± 2	92,336 ± 238
MCS-22	11.5	149.8 ± 0.3	6701 ± 135	306 ± 6	438.5 ± 2.6	0.8307 ± 0.0021	88,908 ± 419	88,071 ± 723	562 ± 3	88,001 ± 723
M2	12.1	138.3 ± 0.2	4046 ± 81	468 ± 9	451.2 ± 2.3	0.8306 ± 0.0018	87,663 ± 360	87,122 ± 523	577 ± 3	87,051 ± 523
MCS-21*	12.9	144.0 ± 0.2	8641 ± 173	234 ± 5	446.0 ± 2.4	0.8499 ± 0.0021	91,192 ± 416	90,079 ± 889	575 ± 3	90,009 ± 889
M3	13.7	141.0 ± 0.3	5057 ± 102	384 ± 8	451.0 ± 2.4	0.8359 ± 0.0028	88,496 ± 491	87,833 ± 677	578 ± 3	87,762 ± 677
MCS-20	14	133.5 ± 0.2	3412 ± 68	545 ± 11	448.4 ± 2.3	0.8453 ± 0.0017	90,233 ± 347	89,760 ± 480	578 ± 3	89,690 ± 480
M4*	14.8	122.4 ± 0.2	1667 ± 33	1062 ± 21	433.4 ± 1.8	0.8780 ± 0.0015	97,170 ± 323	96,917 ± 368	570 ± 2	96,846 ± 368
MCS-19*	15	135.2 ± 0.2	7891 ± 158	229 ± 5	430.7 ± 2.0	0.8107 ± 0.0016	86,529 ± 312	85,427 ± 839	548 ± 3	85,357 ± 839
M5*	15.8	135.6 ± 0.2	1837 ± 37	1463 ± 29	460.3 ± 2.0	1.2019 ± 0.0021	162,291 ± 766	162,062 ± 782	727 ± 3	161,991 ± 782
MCS-18*	16	127.1 ± 0.1	2583 ± 52	1355 ± 27	456.3 ± 1.4	1.6703 ± 0.0024				> 500,000
M6	17.1	123.5 ± 0.1	971 ± 19	1868 ± 37	478.4 ± 1.5	0.8904 ± 0.0013	94,341 ± 260	94,200 ± 278	624 ± 2	94,129 ± 278
MCS-17*	17.5	156.8 ± 0.2	1109 ± 22	3602 ± 72	486.2 ± 1.5	1.5459 ± 0.0023	310,035 ± 2520	309,939 ± 2519	1166 ± 9	309,869 ± 2519
M7	18.5	117.4 ± 0.2	3564 ± 72	479 ± 10	471.7 ± 2.4	0.8821 ± 0.0022	93,721 ± 430	93,172 ± 577	613 ± 3	93,101 ± 577
MCS-16	18.8	139.9 ± 0.2	4132 ± 83	487 ± 10	467.9 ± 2.1	0.8727 ± 0.0016	92,612 ± 332	92,076 ± 502	607 ± 3	92,006 ± 502
M8	20.2	119.5 ± 0.2	1866 ± 37	961 ± 19	483.1 ± 2.2	0.9099 ± 0.0019	96,978 ± 389	96,699 ± 435	635 ± 3	96,628 ± 435
MCS-15	20.5	114.2 ± 0.1	1175 ± 24	1435 ± 29	460.2 ± 1.6	0.8956 ± 0.0013	97,134 ± 279	96,947 ± 308	605 ± 2	96,877 ± 308
M9	21.3	105.3 ± 0.1	173 ± 3	9287 ± 188	509.0 ± 1.5	0.9239 ± 0.0013	96,467 ± 259	96,438 ± 259	668 ± 2	96,367 ± 259
MCS-14	21.8	116.4 ± 0.1	2100 ± 42	822 ± 16	476.9 ± 1.8	0.8990 ± 0.0015	95,877 ± 310	95,553 ± 384	625 ± 3	95,483 ± 384
M10*	22.6	123.4 ± 0.3	6433 ± 129	297 ± 6	498.3 ± 3.0	0.9407 ± 0.0027	100,343 ± 560	99,425 ± 853	660 ± 4	99,354 ± 853
MCS-13	23	150.9 ± 0.2	3707 ± 74	612 ± 12	488.7 ± 2.1	0.9122 ± 0.0017	96,740 ± 354	96,304 ± 468	641 ± 3	96,234 ± 468
M11	24	125.9 ± 0.1	993 ± 20	1939 ± 39	518.9 ± 1.6	0.9281 ± 0.0013	96,094 ± 268	95,957 ± 285	680 ± 2	95,886 ± 285
MCS-12	24.1	143.4 ± 0.1	1771 ± 35	1227 ± 25	497.5 ± 1.5	0.9191 ± 0.0012	96,911 ± 255	96,693 ± 297	654 ± 2	96,623 ± 297
M12	25.1	138.5 ± 0.2	1098 ± 22	1898 ± 38	490.4 ± 1.9	0.9125 ± 0.0014	96,617 ± 304	96,476 ± 319	644 ± 3	96,405 ± 319
MCS-11	25.8	121.5 ± 0.1	3817 ± 76	472 ± 9	476.3 ± 1.8	0.8998 ± 0.0017	96,068 ± 336	95,504 ± 521	624 ± 3	95,434 ± 521
M13	26.1	118.0 ± 0.1	318 ± 6	5525 ± 112	471.1 ± 1.4	0.9043 ± 0.0015	97,370 ± 290	97,322 ± 292	620 ± 2	97,251 ± 292
M14	27.5	98.9 ± 0.1	1009 ± 20	1449 ± 29	468.8 ± 1.8	0.8963 ± 0.0014	96,307 ± 294	96,122 ± 321	615 ± 2	96,051 ± 321
M15	29.4	108.3 ± 0.1	618 ± 12	2567 ± 52	464.1 ± 1.7	0.8887 ± 0.0012	95,573 ± 264	95,470 ± 274	608 ± 2	95,399 ± 274
M16	30.8	95.0 ± 0.1	160 ± 3	8765 ± 178	463.9 ± 1.7	0.8976 ± 0.0013	97,057 ± 284	97,026 ± 284	610 ± 2	96,955 ± 284
MCS-10	31.5	103.8 ± 0.1	1640 ± 33	929 ± 19	457.1 ± 2.1	0.8902 ± 0.0016	96,583 ± 352	96,295 ± 406	600 ± 3	96,225 ± 406
M17	32.1	115.6 ± 0.3	7580 ± 152	224 ± 5	454.2 ± 2.7	0.8893 ± 0.0029	96,742 ± 560	95,540 ± 1014	595 ± 4	95,469 ± 1014
MCS-9*	33.4	103.1 ± 0.1	1884 ± 38	823 ± 17	452.3 ± 1.8	0.9124 ± 0.0015	100,859 ± 337	100,526 ± 410	601 ± 3	100,456 ± 410
M18	33.8	96.2 ± 0.2	2075 ± 42	684 ± 14	452.0 ± 2.5	0.8948 ± 0.0024	97,900 ± 487	97,506 ± 559	595 ± 3	97,435 ± 559
M19	35.5	91.8 ± 0.2	6700 ± 135	205 ± 4	459.4 ± 2.8	0.9084 ± 0.0027	99,354 ± 558	98,025 ± 1089	606 ± 4	97,954 ± 1089
MCS-8	36.4	102.1 ± 0.1	1683 ± 34	882 ± 18	444.1 ± 1.8	0.8815 ± 0.0015	96,566 ± 327	96,262 ± 390	583 ± 2	96,192 ± 390
M20	36.8	95.3 ± 0.2	1838 ± 37	760 ± 15	445.8 ± 2.6	0.8899 ± 0.0020	97,776 ± 448	97,422 ± 512	587 ± 4	97,351 ± 512

*Refers to dating results that were not used in the age model. B.P. stands for “Before Present” where the “Present” is defined as the year 1950 A.D.

**Figure 3:**
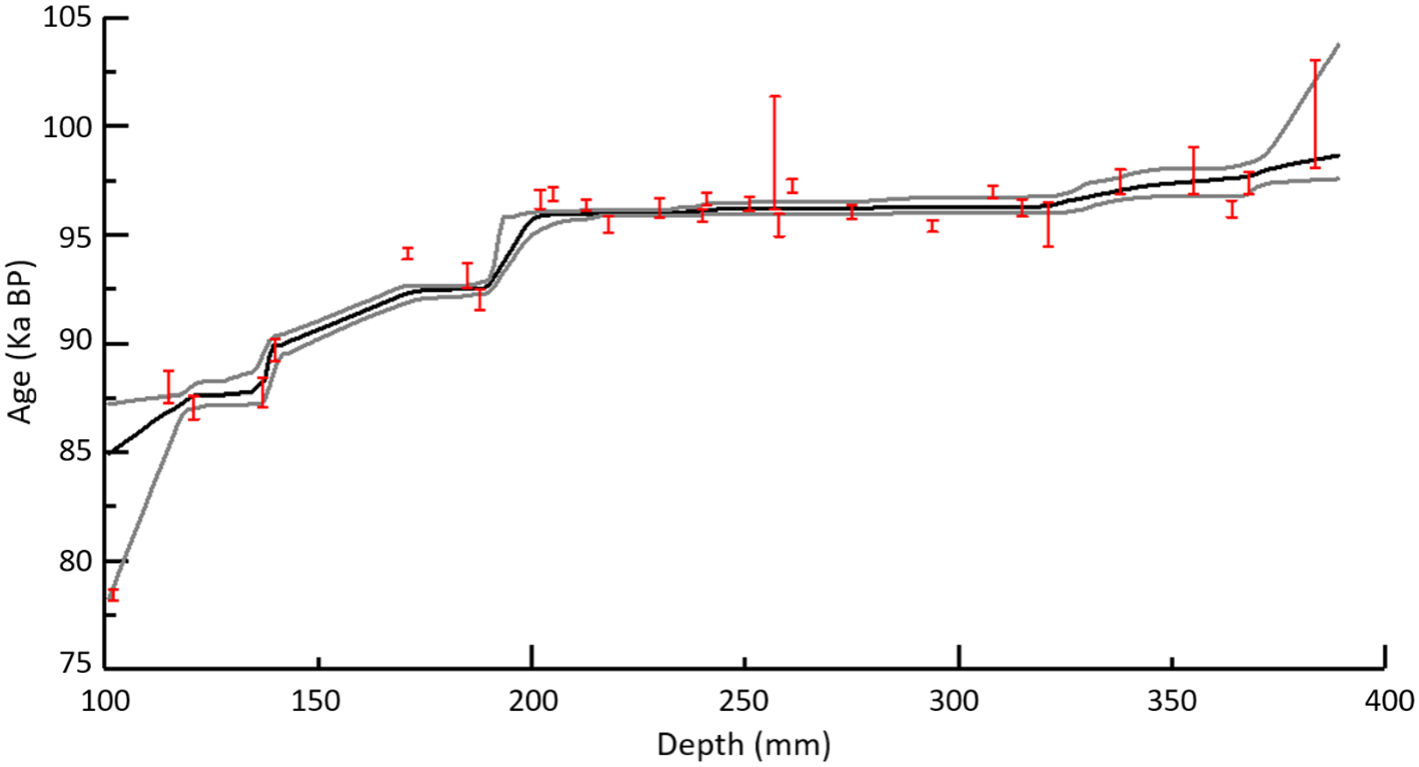
Chronological age-depth model developed for the stalagmite MCS-01 during MIS 5b based on StalAge Algorithm^36^. The grey lines represent the age model’s 95% confidence intervals. Age uncertainties of dated samples used in the age model are shown as well. Minor outliers shown in the age model we still considered as their uncertainty is increased using an iterative procedure by the StalAge Algorithm.

The final age‐depth model was constructed with 33 U/Th ages (including 4 U/Th ages for the Holocene period which is not the main focus of this manuscript), using the StalAge algorithm^36^ (Fig. 3). Ages are reported in years BP with 1950 AD as the year of reference. The growth of the stalagmite was active when it was collected. The U/Th dating results show that the stalagmite had three main growth phases, from 100.5 ± 2.5 to 85 ± 4.2 ka BP, which corresponds to MIS 5b, followed by a growth hiatus of several tens of thousands of years. The growth of the stalagmite was restored from 11 ± 0.25 to 10.5 ± 0.25 ka BP and was interrupted until 0.5 ± 0.1 ka BP.

### Description of δ^18^O and δ^13^C records

**Figure 4:**
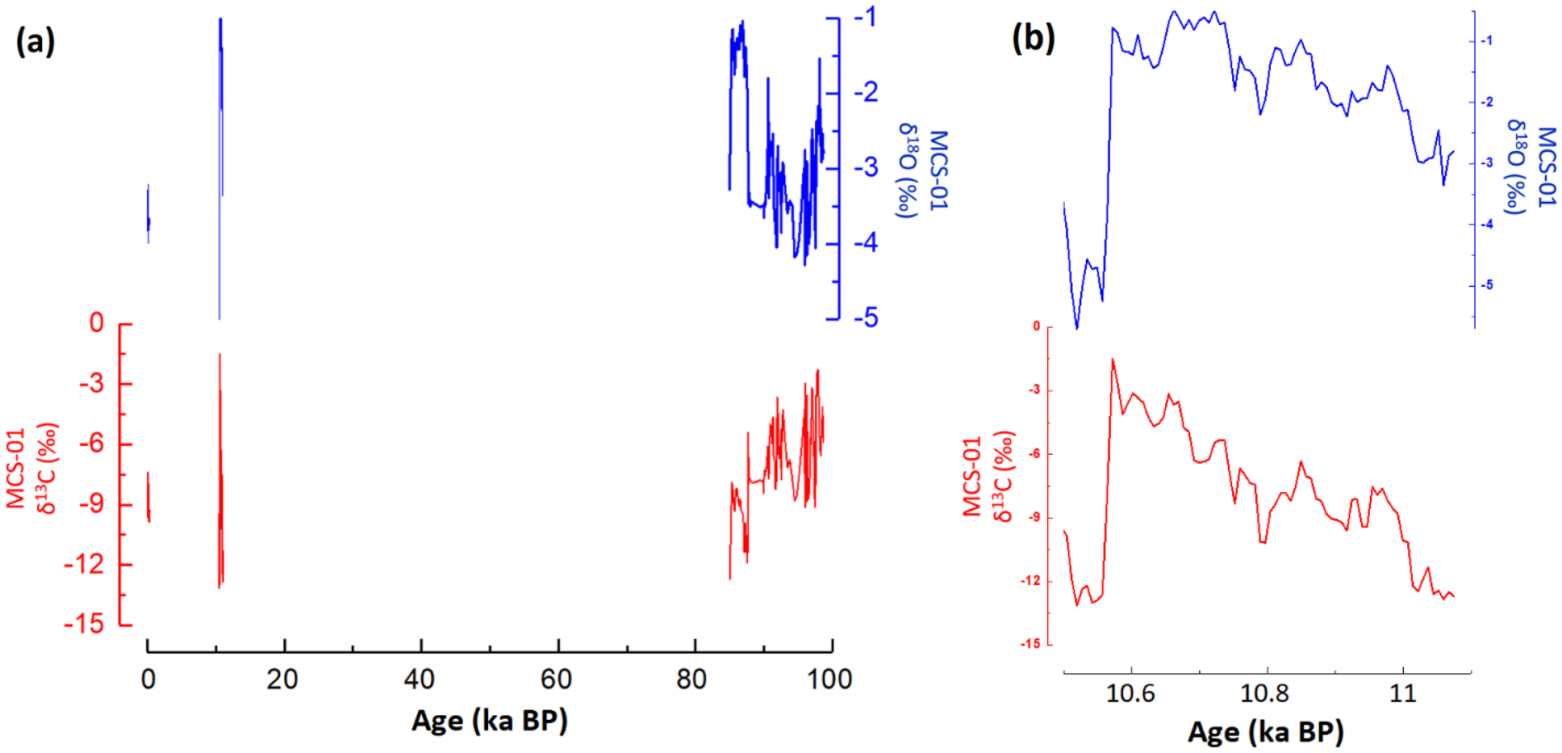
The MCS-01 speleothem isotope (δ^18^O and δ^13^C) records from Majaguas Upper cave in Western Cuba (**a**); the early Holocene record of stalagmite MCS-01 is shown as well (**b**).

The stable isotope results show considerable variability between the growth periods (Holocene vs. MIS 5b) with respect to both the δ^18^O and δ^13^C values (Fig. 4). During MIS 5b, δ^18^O values ranged from − 1.1 to − 4.3‰ and the δ^13^C values ranged from − 2.4‰ to − 11.9‰. Interestingly, the δ^18^O values are consistently low from 90.2 ± 0.4 to 87.6 ± 0.5 ka BP and then undergo an abrupt increase to near − 1‰. The δ^13^C values in this section of the stalagmite, however, respond in an inverse manner, becoming more negative and reaching values of approximately − 10‰. Prior to this, δ^18^O and δ^13^C values show similar peaks and troughs. In the early Holocene section of the stalagmite, δ^18^O values range from − 0.5 to − 5.2‰ and − 1.5 to − 12.9‰ for δ^13^C. This time period has a much greater range and variability compared to MIS5b, and both the δ^18^O and δ^13^C data reveal a consistent increase throughout this period, before undergoing abrupt decreases. For the late Holocene section, the δ^18^O and δ^13^C data are the least variable; these values are − 3.2 to − 5.7‰ and − 7.4 to − 13.1‰ respectively. Previous results in western Cuba revealed that the Caribbean region had a substantial change in temperature^37,38^, confirmed by a more recent speleothem paleoclimate evidence from Puerto Rico, which indicates a cooler last glacial maximum compared to the Holocene^39^.

The analyses of drip water and speleothems from western Cuban caves showed that the δ^18^O values in this area are mainly dominated by the summer rainfall^1^. The δ^18^O data from western Cuba speleothems has been interpreted in recent studies as representing a primarily climatic signal, with more positive δ^18^O values indicative of drier climate conditions, and more negative δ^18^O values in wetter conditions^6,13,40^. The carbon isotopic results from speleothems are more complicated and can be indicative of a number of other processes in addition to climate change, such as vegetation productivity^2^, changes in atmospheric pCO_2_, and hydrological or biological processes in the soil and/or the epikarst. Hence, the remainder of the discussion focuses mostly on the δ^18^O data.

### Local response to North Atlantic abrupt cooling events H8 and H9

**Figure 5:**
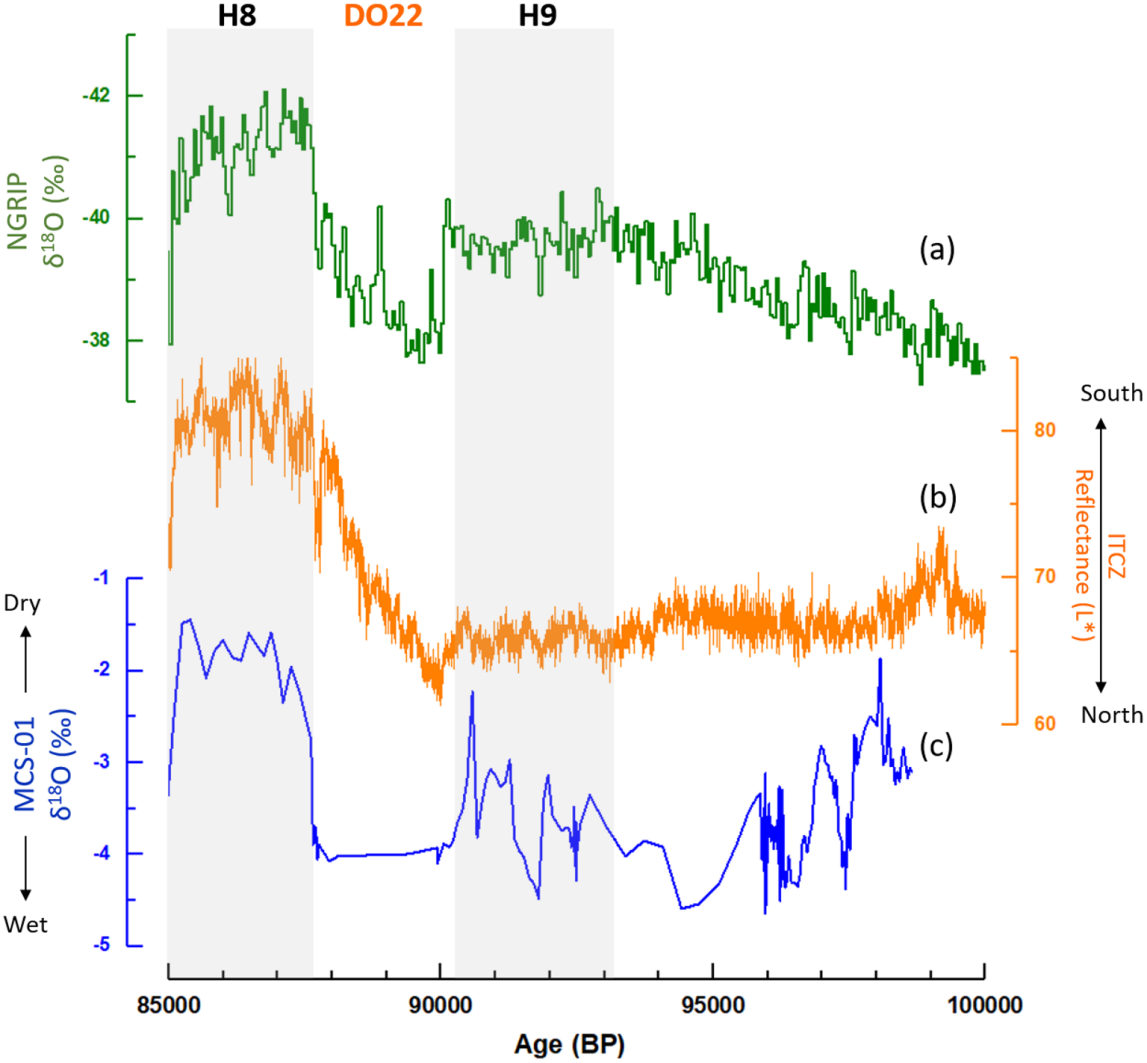
Comparison of the MCS-01 speleothem δ^18^O record from Majaguas Upper cave in western Cuba (**c**) with the North Greenland Ice Core Project (**a**)^23–28^ and the reflectance record from the sediment core MDO3-2621 from the Cariaco Basin (**b**) representing ITCZ movements^17^.

The MCS-01 record covers the period between 98.7 ± 3.1 and 84.9 ± 4.5 ka BP within MIS 5b and two relatively short periods from 11.1 ± 0.25 to 10.5 ± 0.25 ka BP and from 0.4 ± 0.1 to present 0.05 ± 0.7 ka BP. Hence, our discussion in this paragraph will focus on the MIS 5b. The δ^18^O record was corrected for global ice volume changes by subtracting 0.008‰ per meter of sea-level change^41^, using the most detailed sea level data with an independent age model by Grant et al.^42^. The corrected record presents δ^18^O values ranging between - 1 and − 4.3‰, with three distinctive periods of high δ^18^O corresponding to dry periods 87.6 ± 0.5 to 85 ± 4.2, 93.1 ± 0.5 to 90.2 ± 0.4, and 98.6 ± 2.9 to 95.9 ± 0.3 ka BP. We compare the MCS-01 δ^18^O data (Fig. 5c) to the ITCZ record from the Cariaco Basin (Fig. 5b)^17^ and the NGRIP ice core (Fig. 5a)^23–28^ to assess a tropical response to high northern latitude climatic changes.

The dry and/or cold periods of 87.6 ± 0.5 to 85 ± 4.2 and 93.1 ± 0.5 to 90.2 ± 0.4 ka BP seem to be synchronous with Heinrich events 8 and 9 (H8 and H9) and corresponding southward shifts of the ITCZ at those times. Interestingly, the H8 event seems more pronounced in western Cuba compared to H9, with persistent positive δ^18^O values which are the highest in the MCS-01 record. This can be explained by North Atlantic temperatures, which were colder during H8 compared to H9 as shown by the NGRIP record, and the stronger southward shift of the ITCZ. These relationships represent the bipolar seesaw in which cold conditions in the North Atlantic during H8 and H9 might have been linked via the thermohaline circulation to warm conditions in the southern hemisphere, such that cold conditions in the North Atlantic are associated with a southward shift of ITCZ, and hence dry and/or colder conditions in Cuba. This is a new result showing for the first time a local climate response in Western Cuba to H8 and H9. However, caution is still needed given the large age model uncertainties around 85 ka BP.

**Figure 6:**
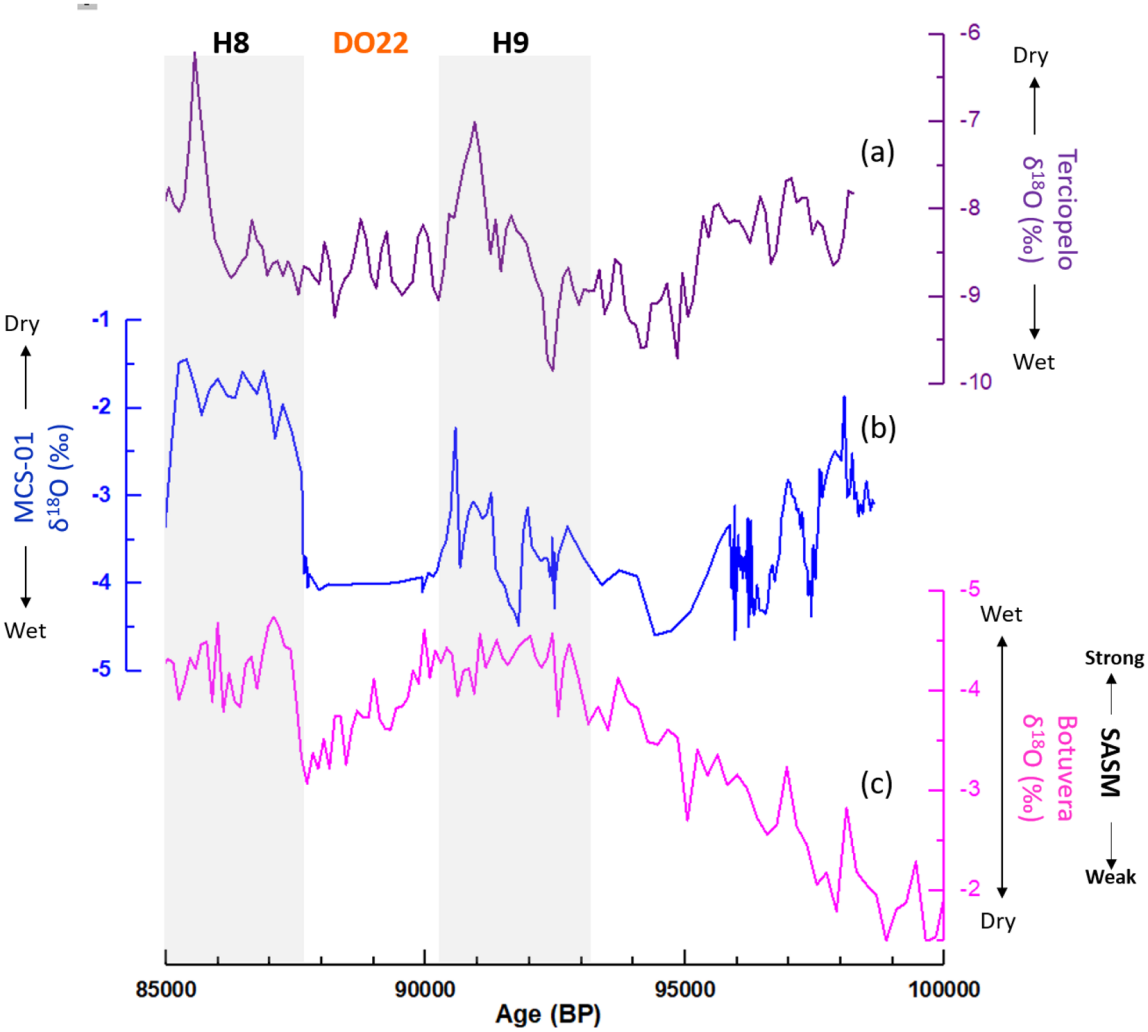
Comparison of the MCS-01 speleothem δ^18^O record from Majaguas Upper cave in western Cuba (**b**) with the Terciopelo speleothem record from Central America (**a**)^15^ and the Botuvera speleothem record from SE Brazil (**c**)^22^.

The North Atlantic cold events H8 and H9 were characterized by cooler and/or drier conditions not only in Cuba, but also in Central America (CA), as shown by a speleothem record from Costa Rica^15^ (Fig. 6a). The close connection between Cuba and Costa Rica derives from the easterly trade winds associated with the North Atlantic Subtropical High, which strengthen the Caribbean Low-Level Jet (CLLJ), and drive moisture towards both the western Gulf of Mexico and the CA isthmus to the Pacific^13^. Speleothem records from Western Cuba and Central America show considerable similarities during North Atlantic abrupt climate events^13,15,17^. Furthermore, the comparison of our data (Fig. 6b) with the Botuvera speleothem record from SE Brazil^22^ (Fig. 6c) reinforces the idea of anti-phased climate conditions between the Caribbean and South America caused by ITCZ shift at the millennial timescale. For instance, a southern position of the ITCZ observed during H8 leads to significant dry conditions in the Caribbean while it corresponds to a stronger SAMS regime to the south. Indeed, the highest δ^18^O values in the MCS-01 record are found during H8 between (~ 1‰). The Greenland Interstadial (Dansgaard-Oeschger; DO) event 22 is also associated with low δ^18^O values which suggest a northward shift of the ITCZ and the prevalence of wet conditions in the Caribbean, whereas dry conditions are established in the SASM domain, consistent with Strikis et al.^43^.

### Ocean and atmospheric mechanisms during the Last Glacial period

In terms of comparisons to other local studies, the Cuba Medio (CM) speleothem record from western Cuba dates to MIS 5^13^, but CM is mostly discontinuous though the time period covered by stalagmite MCS-01. The comparison with the CM record shows that the absolute δ^18^O values are not the same during the contemporary growth period of both stalagmites from 96.2 ± 0.25 to 92.8 ± 0.3 ka BP, whereas the CM record presents higher absolute δ^18^O values. This can be explained by drip water and site-specific conditions^15^. For instance, the CM stalagmite was collected in Torch Cave (western Cuba) at 170 m above sea level, which is overlain by approximately 60 m of rock^13^. This is in contrast to the ~ 100 m of rock overlying stalagmite MCS-01. However, both speleothem records show a similar trend with a transition from low to high δ^18^O values between 96.2 ± 0.25 and 92.8 ± 0.3 ka BP indicating a resumption of drier conditions during the onset of H9.

**Figure 7:**
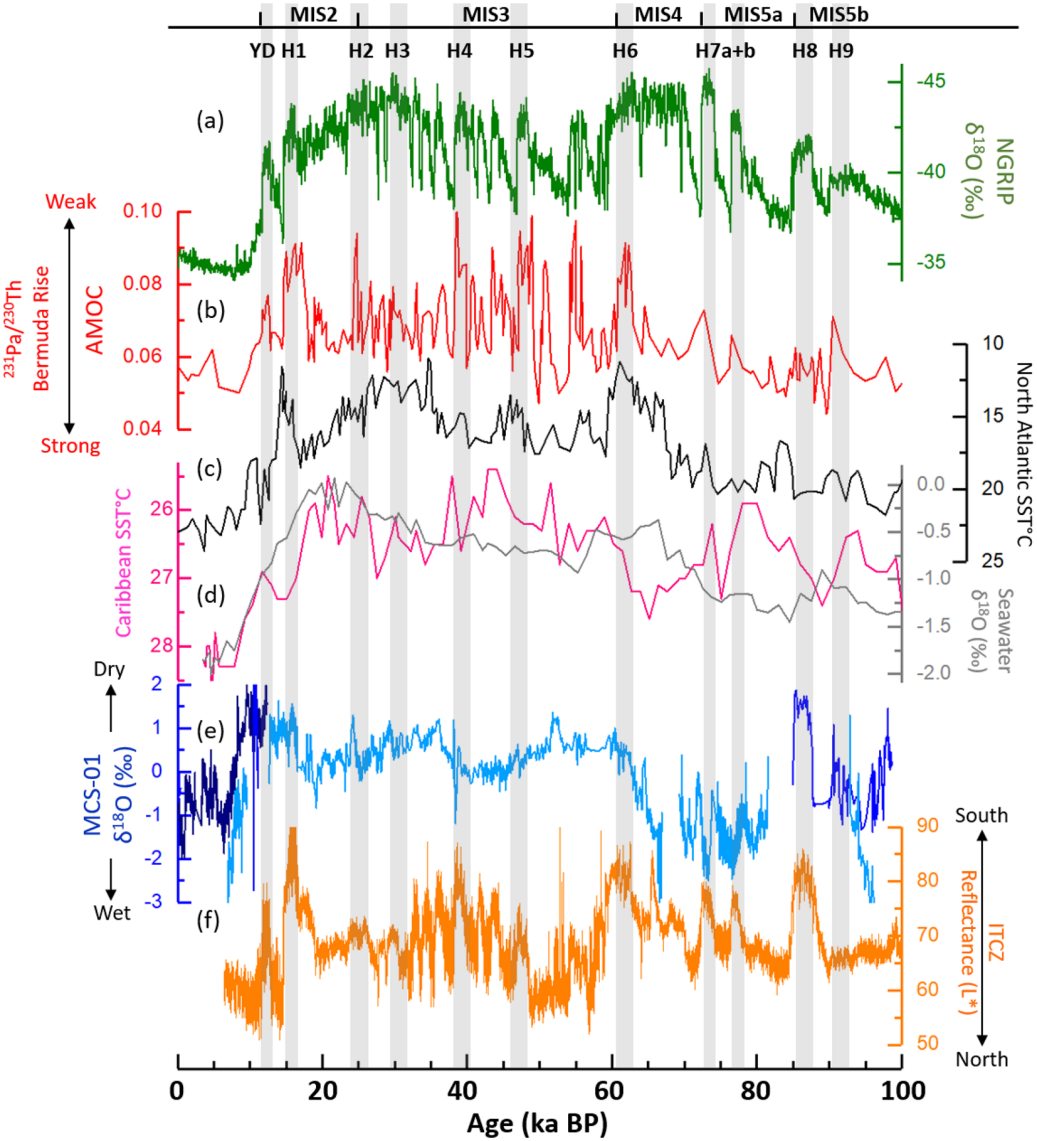
Comparison of the Western Cuban speleothem records (normalized data) (e) by Fensterer et al.^6^ (dark blue), Warken et al.^13^ (light blue) and this study (blue), with the North Greenland Ice Core Project (**a**)^23–28^, the Bermuda Rise ^231^ Pa/^230^Th as a proxy of AMOC strength (**b**)^18,20,21^, the North Atlantic SST from core SU90-03 (**c**)^48^, the Caribbean Sea Surface Temperature (SST) and δ^18^O records (**d**) inferred from planktonic foraminifera from the sediment core ODP 999A^16^, and the reflectance record from the sediment core MDO3-2621 (**f**) from the Cariaco Basin reflecting ITCZ movements^17^. Gray bars indicate the timing of the North Atlantic abrupt cooling episodes (Heinrich events).

Other than the CM record of Warken et al.^13^, we also compare our MCS-01 results to multi-speleothem records of δ^18^O values from the Cuba Pequeño (CP) data from the Dos Anas cave system^6^ (Fig. 7e). The western Cuban multi-speleothem record (composite of MCS-01, CP and CM speleothem records) covers the last 100 ka at high resolution and allows us to assess the mechanisms that modulated rainfall variability in the region during the last glacial period through comparison with regional paleoclimate data and variability modes. On orbital timescales, the multi-speleothem δ^18^O record shares similarities with the ITCZ reconstruction (Fig. 7f) and the SST records in the Caribbean basin (Fig. 7d)^16^ with higher rainfall in western Cuba during periods of warm tropical North Atlantic Ocean. The multi-speleothem record is consistent with winter insolation on glacial-interglacial timescales^13^. Indeed, enhanced winter insolation may induce a warming effect on surface waters in the Caribbean, which would result in an extended humid season^15,44^.

Previous work suggests a close relationship between the Caribbean and the North Atlantic climate during the last glacial period^13^. Positive peaks in Western Cuban speleothem δ^18^O records are synchronous with the Heinrich events (Fig. 7a). Furthermore, a remarkable consistency was observed with the Bermuda rise ^231^ Pa/^230^Th record of AMOC strength^18,20,21^, whereas the positive peaks in the speleothem record correspond to dry events, which coincide with cooler temperatures in the North Atlantic and periods of reduced AMOC during stadials^13^ (Fig. 7b). Indeed, the Heinrich events are associated with strong freshwater flow in the North Atlantic Ocean^45,46^, which resulted in the weakening of the AMOC due to reduced deep water formation^18,20,47^. Interestingly, the climate response in Western Cuba to H8 seems relatively as severe as during the mega-drought period of H1, as shown by the range of variability in the speleothem δ^18^O values. However, this is not particularly observed in the AMOC strength records nor in the North Atlantic SST^48^ (Fig. 7c) and the NGRIP records, whereas this phenomenon is prominent the ITCZ record which indicates a substantial southward shift of the ITCZ during both H8 and H1 events (Fig. 7f). Hence, the abrupt changes observed during both events are limited to the Western Cuban region and might have been amplified by the ITCZ changes.

## Conclusion

The Caribbean is a key region to obtain information about past natural climate variability in the tropics and its relationship to climate forcing mechanisms. However, paleoclimate evidence from this region is still scattered and limited. In this study, we presented a new speleothem record from Majaguas Upper cave in Western Cuba. The MCS-01 record covers the period between 98.7 ± 3.1 and 84.9 ± 4.5 ka BP within MIS 5b, which was not documented before in Cuba.

The comparison of our data with the NGRIP ice core record showed that two distinct dry and/or cold periods of 87.6 ± 0.5 to 85 ± 4.2 and 93.1 ± 0.5 to 90.2 ± 0.4 ka BP are synchronous with the Heinrich events 8 and 9. The H8 event is more pronounced in western Cuba compared to H9, associated with persistent positive δ^18^O values which are the highest in our record. This is associated with cooler sea surface temperatures in the North Atlantic Ocean during H8 as compared to H9. Our results are consistent with the stronger southward shift of the ITCZ during H8 from the Cariaco Basin record, providing crucial information to fill the gaps of previous speleothem records from the region. Hence, a multi-speleothem record from Western Cuba spanning the last 100 ka with a high resolution reveals a consistent response to Heinrich events, as positive peaks in speleothem δ^18^O records indicate dry and/or cooler conditions in the Caribbean as a response to the weakening of the AMOC due to reduced deep water formation resulting from cooler sea surface temperatures in the North Atlantic high latitudes.

In summary, as compared with regional paleoclimate records, we show that the North Atlantic cold events H8 and H9 were characterized by cooler and/or drier conditions not only in Cuba but also in Central America, which resulted from easterly trade winds associated with the North Atlantic Subtropical High, that strengthen the Caribbean Low-Level Jet and drive the moisture towards both the western Gulf of Mexico and the CA isthmus to the Pacific. Moreover, our data, as compared with South American paleoclimate evidence, reinforce the idea of anti-phased climate conditions between the Caribbean and South America caused by the ITCZ shift during millennial-scale events. Therefore, we suggest that a more southward position of the ITCZ observed during H8 and H9 leads to significant dry conditions in the Caribbean whereas it corresponds to a stronger SAMS regime to the south.

## Data Availability

The dataset produced in this study has been uploaded to the NOAA Paleoclimate Database and can be accessed via this link: https://www.ncei.noaa.gov/access/paleo-search/study/37098.

## References

[1] Fensterer, C. (2011). *Holocene Caribbean Climate Variability reconstructed from Speleothems from Western Cuba*. (PhD. Thesis). University of Heidelberg, Germany.

[2] Fensterer C, Scholz D, Hoffmann D, Spötl C, Pajón JM, Mangini A (2012). Cuban stalagmite suggests relationship between Caribbean precipitation and the Atlantic multidecadal oscillation during the past 1.3 ka. Holocene.

[3] Hodell D, Curtis J, Brenner M (1995). Possible role of climate in the collapse of classic Maya civilization. Nature.

[4] Medina-Elizalde M, Burns SJ, Lea DW, Asmerom Y, von Gunten L, Polyak V, Vuille M, Karmalkar A (2010). High resolution stalagmite climate record from the Yucatán Peninsula spanning the Maya terminal classic period. Earth Planet. Sci. Lett..

[5] Lane CS, Horn SP, Kerr MT (2014). Beyond the Mayan lowlands: impacts of the terminal classic drought in the Caribbean Antilles. Quat. Sci. Rev..

[6] Fensterer C, Scholz D, Hoffmann DL, Spötl C, Schröder-Ritzrau A, Horn C, Pajón JM, Mangini A (2013). Millennial-scale climate variability during the last 12.5 ka recorded in a Caribbean speleothem. Earth Planet. Sci. Lett..

[7] Gregory BRB, Peros M, Reinhardt EG, Donnelly JP (2015). Middle–late holocene Caribbean aridity inferred from foraminifera and elemental data in sediment cores from two Cuban lagoons. Palaeogeogr. Palaeoclimatol. Palaeoecol..

[8] Peros MC, Reinhardt EG, Schwarcz HP, Davis AM (2007). High-resolution paleosalinity reconstruction from Laguna de la Leche, north coastal Cuba, using Sr, O, and C isotopes. Palaeogeogr. Palaeoclimatol. Palaeoecol..

[9] Peros M, Collins S, G’Meiner AA, Reinhardt E, Pupo FM (2017). Multistage 8.2 kyr event revealed through high-resolution XRF core scanning of Cuban sinkhole sediments: Sinkhole sediments reveal 8.2 kyr event. Geophys. Res. Lett..

[10] Peros MC, Gregory B, Matos F, Reinhardt E, Desloges J (2015). Late-Holocene record of lagoon evolution, climate change, and hurricane activity from southeastern Cuba. Holocene.

[11] Pajón JM, Hernández I, Ortega F, Macle J, Markgraf V (2001). Periods of wet climate in Cuba: Evaluation of expression in karst of Sierra de San Carlos. Interhemispheric Climate Linkages.

[12] Schielein P, Christoph Burow C, Pajón JM, Rojas-Consuegra R, Zhao J, Schellmann G (2020). ESR and U-Th dating results for last interglacial coral reef terraces at the northern coast of Cuba. Quatern. Int..

[13] Warken S, Scholz D, Spotl C, Jochum KP, Pajon JM, Bahr A, Mangini A (2019). Caribbean hydroclimate and vegetation history across the last glacial period. Quat. Sci. Rev..

[14] Fairchild IJ, Baker A, Asrat A (2012). Speleothem Science: From Process to Past Environments.

[15] Lachniet MS, Johnson L, Asmerom Y, Burns SJ, Polyak V, Patterson WP, Burt L, Azouz A (2009). Late quaternary moisture export across central America and to Greenland: Evidence for tropical rainfall variability from Costa Rican stalagmites. Quat. Sci. Rev..

[16] Schmidt MW, Spero HJ, Lea DW (2004). Links between salinity variation in the Caribbean and North Atlantic thermohaline circulation. Nature.

[17] Schneider T, Bischoff T, Haug GH (2014). Migrations and dynamics of the intertropical convergence zone. Nature.

[18] McManus JF, Francois R, Gherardi J-M, Keigwin LD, Brown-Leger S (2004). Collapse and rapid resumption of Atlantic meridional circulation linked to deglacial climate changes. Nature.

[19] Channell JF, Hodell R, Curtis J-M (2012). Odp site 1063 (Bermuda rise) revisited: oxygen isotopes, excursions and paleointensity in the brunhes chron. Geochem. Geophys. Geosy.

[20] Bohm E, Lippold J, Gutjahr M, Frank M, Blaser P, Antz B, Fohlmeister J, Frank N, Andersen M, Deininger M (2015). Strong and deep atlantic meridional overturning circulation during the last glacial cycle. Nature.

[21] Henry L, McManus JF, Curry WB, Roberts NL, Piotrowski AM, Keigwin LD (2016). North Atlantic ocean circulation and abrupt climate change during the last glaciation. Science.

[22] Cruz F, Burns S, Karmann I, Sharp W, Vuille M, Cardoso A, Ferrari J, Dias P, Viana O (2005). Insolation-driven changes in atmospheric circulation over the past 116,000 years in subtropical Brazil. Nature.

[23] NGRIP (2004). High-resolution record of Northern Hemisphere climate extending into the last interglacial period. Nature.

[24] Vinther BM, Clausen HB, Johnsen SJ, Rasmussen SO, Andersen KK, Bouchardt SL, Dahl-Jensen D, Seierstad IK, Siggard-Andersen M-L, Steffensen JP, Svensson A, Olson J, Heinemeier J (2006). A synchronized dating of three Greenland ice cores throughout the Holocene. J. Geophys. Res.: Atmos..

[25] Rasmussen SO, Andersen KK, Svensson AM, Steffensen JP, Vinther BM, Clausen HB, Siggaard-Andersen M-L, Johnsen SJ, Larsen LB, Dahl-Jensen D, Bigler M, Röthlisberger R, Fischer H, Goto-Azuma K, Hansson ME, Ruth U (2006). A new Greenland ice core chronology for the last glacial termination. J. Geophys. Res.: Atmos..

[26] Andersen KK, Svensson A, Johnsen SJ, Rasmussen SO, Bigler M, Röthlisberger R, Ruth U, Siggaard-Andersen M-L, Steffensen JP, Dahl-Jensen D, Vinther BM, Clausen HB (2006). The Greenland ice core chronology 2005, 15–42 ka. Part 1: Constructing the time scale. Quat. Sci. Rev..

[27] Svensson A, Andersen KK, Bigler M, Clausen HB, Dahl-Jensen D, Davies SM, Johnsen SJ, Muscheler R, Parrenin F, Rasmussen SO, Rothlisberger R, Seierstad I, Steffensen JP, Vinther BM (2008). A 60,000 year Greenland stratigraphic ice core chronology. Clim. Past Eur. Geosci. Union (EGU).

[28] Wolff EW, Chappellaz J, Blunier T, Rasmussen SO, Svensson A (2010). Millennial-scale variability during the last glacial: The ice core record. Quatern. Sci. Rev..

[29] Peel MC, Finlayson BL, McMahon TA (2007). Updated world map of the Köppen-Geiger climate classification. Hydrol. Earth Syst. Sci..

[30] Pajón, J. M., Curtis, J., Tudhope, S., Metcalfe, S., Brenner, M., Guilderson, T., Chicott, C., Grimm, E., Hernández, I. (2006). Isotope records from a stalagmite from Dos Anas cave in Pinal Del Rio Provience, Cuba. Palaeoclimatic Implications. International Symposium on Nuclear & Related Techniques.

[31] Ponte JM, Font E, Veiga-Pires C, Hillaire-Marcel C, Ghaleb B (2017). The effect of speleothem surface slope on the remanent magnetic inclination: Speleothem shape and magnetism. J. Geophys. Res.: Solid Earth.

[32] Edwards RL, Chen JH, Wasserburg GJ (1987). 238U–234U- 230Th-232Th systematics and the precise measurement of time over the past 500,000 years. Earth Planet Sci. Lett..

[33] Cheng H, Edwards R, Hoff J, Gallup C, Richards D, Asmerom Y (2000). The half lives of uranium-234 and thorium-230. Chem. Geol..

[34] Cheng H, Edwards RL, Shen C-C, Polyak VJ, Asmerom Y, Woodhead J, Hellstrom J, Yongjin W, Kong X, Spotl C, Wang X, Calvin Alexander E (2013). Improvements in Th-230 dating, Th-230 and U-234 half-life values, and U-Th isotopic measurements by multicollector inductively coupled plasma mass spectrometry. Earth Planet Sci. Lett..

[35] Lachniet MS, Bernal JP, Asmerom Y, Polyak V (2012). Uranium loss and aragonite–calcite age discordance in a calcitized aragonite stalagmite. Quat. Geochronol..

[36] Scholz D, Hoffmann DL (2011). Stalage: An algorithm designed for construction of speleothem age models. Quat. Geochronol..

[37] Pajón, J.M., Fundora, M., Pedroso, I., y Jaimez, E., (2003). Paleoregistros naturales isotópicos, paleomagnéticos y edáficos, indicadores de cambios climáticos en Cuba Occidental durante el Cuaternario. MEMORIAS GEOINFO´2003, LA HABANA, 24–28 de Marzo. ISBN 959-7117-11-8. GOGC-06, pp. 30–45.

[38] Pajón, J. M., (2007). Cambios Climáticos Abruptos en la Transición Pleistoceno-Holoceno a partir de Paleoregistros Isotópicos. Casos de estudio con espeleotemas. CD Rom Segunda Convención Cubana de Ciencias de la Tierra. Palacio de Convenciones. La Habana, Cuba, Marzo 20–23/2007, 12 pp. ISBN 978-959-7117-16-2.

[39] Warken SF, Weißbach T, Kluge T, Vonhof H, Scholz D, Vieten R, Schmidt M, Winter A, Frank N (2022). Last glacial millennial-scale hydro-climate and temperature changes in Puerto Rico constrained by speleothem fluid inclusion δ18O and δ2H values. Clim. Past.

[40] Oster JL, Montanez IP, Guilderson TP, Sharp WD, Banner JL (2010). Modeling speleothem d13c variability in a central sierra Nevada cave using 14c and 87sr/ 86sr. Geochem. Cosmochim. Acta.

[41] Duplessy JC, Roche DM, Kageyama M (2007). The deep ocean during the last interglacial period. Science.

[42] Grant KM, Rohling EJ, Bar-Matthews M, Ayalon A, Medina-Elizalde M, Bronk RC, Satow C, Roberts AP (2012). Rapid coupling between ice volume and polar temperature over the past 150,000 years. Nature.

[43] Stríkis MN, Cruz FW, Barreto EAS, Naughton F, Vuille M, Cheng H, Voelker AHL, Zhang H, Karmann I, Edwards RL, Auler AS, Santos RV, Sales HR (2018). South American monsoon response to iceberg discharge in the North Atlantic. PNAS.

[44] Enfield DB, Alfaro EJ (1999). The dependence of caribbean rainfall on the interaction of the tropical atlantic and pacific oceans. J. Clim..

[45] Heinrich H (1988). Origin and consequences of cyclic ice rafting in the northeast Atlantic Ocean during the past 130,000 years. Quat. Res..

[46] Hemming SR (2004). Heinrich events: massive late pleistocene detritus layers of the North Atlantic and their global climate imprint. Rev. Geophys..

[47] Medina-Elizalde M, Burns SJ, Polanco-Martinez J, Lases-Hernández F, Bradley R, Wang H, Shen C (2017). Synchronous precipitation reduction in the American tropics associated with Heinrich 2. Sci. Rep..

[48] Chapman MR, Shackleton NJ, Duplessy J-C (2000). Sea surface temperature variability during the last glacial-interglacial cycle: assessing the magnitude and pattern of climate change in the North Atlantic. Palaeogeogr. Palaeoclimatol. Palaeoecol..

